# Performance of federated versus centralized learning for mammography classification across film–digital domain shift

**DOI:** 10.3389/fdgth.2026.1715858

**Published:** 2026-03-25

**Authors:** Yaqeen Ali, Julia Müller, Andreas Weinmann, Johannes Gregori

**Affiliations:** 1 Doctoral Center Applied Informatics, University of Applied Sciences, Darmstadt, Germany; 2 R&D, mediri GmbH, Heidelberg, Germany; 3 Department of Mathematics and Natural Sciences, University of Applied Sciences, Darmstadt, Germany; 4 Algorithms for Computer Vision, Imaging and Data Analysis Lab, Technische Hochschule Würzburg-Schweinfurt, Schweinfurt, Germany; 5 European University of Technology (EUt+), European University Alliance, European Union

**Keywords:** federated learning, mammography, domain generalization, deep learning, FedAvg, FedProx, SCAFFOLD, FedBN

## Abstract

**Introduction:**

Large, diverse datasets are essential for reliable deep learning in mammography, yet clinical data remain siloed due to privacy and governance constraints. Federated learning enables collaborative training without sharing raw data, but its robustness under strong imaging-domain heterogeneity, such as film–digital shifts, remains uncertain.

**Methods:**

We conducted a comparative evaluation of centralized learning and cross-silo federated learning for benign-malignant lesion classification across two heterogeneous public datasets: CBIS-DDSM (scanned film) and VinDr-Mammo (full-field digital). Using ResNet-50 and Swin V2-T backbones, we evaluated FedAvg, FedProx, SCAFFOLD, and FedBN across multi-seed experiments with bootstrap confidence intervals. The study design included local-only baselines, homogeneous FL controls, size-balancing ablations, and a resolution ablation (224→324 px). Performance was assessed using AUROC, AP, Accuracy, Precision, Recall, F1, and Precision@Recall = 0.90.

**Results:**

Federated models matched centralized learning in homogeneous settings for both domains. Under film-digital heterogeneity, FL retained strong performance on the digital VinDr domain (AUC ≈ 0.91-0.95) but showed reduced performance on the film-based CBIS domain (AUC ≈ 0.53-0.62), exhibiting a shift toward high-recall/low-precision behavior. Neither FedProx, SCAFFOLD, nor FedBN consistently mitigated this degradation. Size-balancing improved CBIS performance modestly but did not close the gap to centralized learning, indicating that feature and quality shift dominated over dataset-size imbalance. Higher input resolution improved CBIS calcification detection (e.g., F1 0.49 → 0.54).

**Discussion:**

These findings show that FL performs reliably within homogeneous domains but remains vulnerable to strong feature and quality shifts between film and digital mammography. The observed asymmetric performance suggests that domain shift, rather than data quantity or optimizer instability, is the dominant limiting factor.

**Conclusion:**

Federated learning enables high-performing mammography classification without data centralization in homogeneous settings but requires domain-aware or personalized FL strategies, site-specific thresholding, and resolution-sensitive preprocessing to ensure reliable deployment under film–digital heterogeneity.

## 1 Introduction

Breast cancer remains the leading cause of cancer-related mortality among women worldwide, and early detection through screening mammography is central to reducing mortality and improving treatment outcomes (1, 2). Advances in deep learning (DL), particularly convolutional neural networks (CNNs) (3) and Vision Transformers (4), have enabled automated systems capable of achieving radiologist-level performance when trained on sufficiently large and diverse datasets (5). However, access to such datasets is severely limited by privacy regulations, ethical constraints, and institutional data-governance policies that prevent cross-site data sharing (6).

Under conventional centralized learning (CL), data must be aggregated into a central repository, placing CL in direct tension with regulations such as HIPAA and GDPR (7, 8). Federated learning (FL) (9) offers an alternative paradigm in which model training occurs locally at each site, and only parameter updates—not raw images—are exchanged. This preserves data sovereignty while enabling collaborative model development. Prior work has shown that FL can approach the performance of CL in relatively homogeneous collaborations (10, 11). Yet real-world clinical imaging networks are rarely homogeneous: differences in scanner type, acquisition parameters, patient populations, and annotation styles often create substantial *feature shift* and *quality skew* across institutions. Such heterogeneity is a principal challenge for FL, as it can lead to client drift, unstable convergence, and degraded global performance (12).

In mammography, the contrast between historical scanned-film studies and modern full-field digital mammography (FFDM) represents a particularly pronounced domain shift. Film images differ from digital images in noise texture, dynamic range, spatial resolution, and digitization artifacts, all of which affect lesion appearance, especially microcalcifications. Although film mammography is now uncommon in high-income screening programs, film–digital heterogeneity remains clinically relevant as a proxy for broader multi-vendor, multi-protocol differences present even in all-digital networks. As such, film–digital contrast provides a controlled yet realistic stress test for evaluating FL under severe non-IID conditions (13, 14).

ResNet-50 (3) and Swin V2-T (4) were chosen as representative CNN and Transformer backbones because both have shown strong performance in recent mammography studies. ResNet-50 has been widely validated for detecting masses and microcalcifications on screening mammograms (15–17), while Swin-based models have achieved competitive results by capturing long-range dependencies and the hierarchical breast-tissue structure (18, 19). These architectures therefore provide complementary inductive biases that are well suited for evaluating lesion-level classification under cross-domain heterogeneity.

To our knowledge, this work provides an integrated evaluation of FL for mammography classification under strong cross-domain heterogeneity, combining: (i) two architecturally distinct datasets (CBIS-DDSM and VinDr-Mammo) (20, 21), (ii) two backbone families (ResNet-50 and Swin Transformer), (iii) four FL algorithms (FedAvg, FedProx, SCAFFOLD, FedBN) (9, 22–24), (iv) robust multi-seed training with bootstrap confidence intervals, and (v) ablations isolating the contributions of quantity imbalance, feature shift, and input resolution. We additionally include local-only baselines to quantify the benefit of collaboration for each client and to contextualize global performance.

We address four research questions:

1. **RQ1: Homogeneous performance.** How closely can FL match CL when training and testing occur within the same domain?
2. **RQ2: Heterogeneous robustness.** How do FedAvg, FedProx, SCAFFOLD, and FedBN compare under a two-client film–digital split, and can any method mitigate minority-domain degradation?
3. **RQ3: Source of performance gaps.** Are observed failures driven primarily by feature/quality shift or by dataset-size imbalance?
4. **RQ4: Clinical operating points and resolution.** How does domain shift affect precision–recall behavior, and whether site-specific thresholding or higher-resolution inputs can improve clinical utility?

By integrating architectural comparisons, heterogeneity-aware FL baselines, rigorous statistical evaluation, and clinically oriented operating-point analyses, this study provides a comprehensive assessment of FL’s potential and limitations for safe deployment in breast cancer screening.

## 2 Materials and methods

### 2.1 Datasets and cohorts

**Figure 1:**
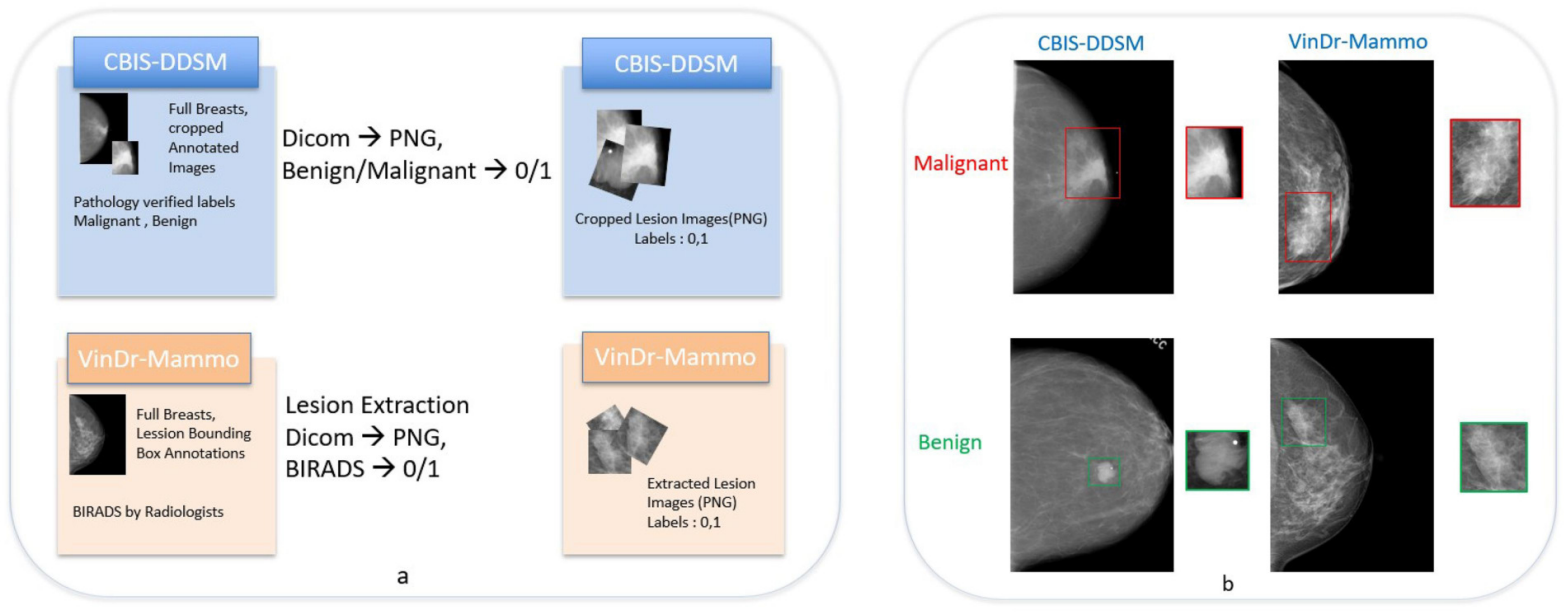
**(a)** Data preparation pipeline. CBIS-DDSM lesion-centered ROIs are used as released; VinDr-Mammo ROIs are obtained by applying provided bounding boxes to the original DICOMs. All ROIs are exported to PNG, preserving pixel intensities at extraction time; resizing to the network input resolution (default 224×224) occurs only at training/inference. BI-RADS labels are mapped to binary classes (0 = benign, 1 = malignant). **(b)** Representative annotated examples from CBIS-DDSM (left column) and VinDr-Mammo (right column); top row: malignant, bottom row: benign. Red boxes: malignant; green boxes: benign.

We studied lesion-level benign/malignant classification under pronounced cross-domain heterogeneity using two public datasets: CBIS-DDSM (scanned film) and VinDr-Mammo (full-field digital mammography; FFDM). Figure 1 illustrates the data preparation pipeline and representative examples from both domains. Table 1 summarizes patient-level splits and class proportions, highlighting quantity and label skew between the two sources (VinDr is larger and more benign-skewed than CBIS). The film–digital contrast is known to induce distinct image statistics (dynamic range, noise/grain, digitization artifacts) that affect lesion appearance especially microcalcifications providing a controlled yet realistic stress test for FL under non-IID conditions.

**Table 1:** Patient-level dataset statistics for CBIS-DDSM and VinDr-Mammo.

Dataset split	Images	Benign (0)%	Malignant (1)%
CBIS (Train + Validation)	2,864	1,683 (58.8%)	1,181 (41.2%)
VinDr (Train + Validation)	16,370	14,463 (88.35%)	1,907 (11.64%)
Total VinDr + CBIS (Train + Validation)	19,234	16,146 (83.94%)	3,088 (16.06%)
CBIS (Test)	1,403	855 (60.94%)	548 (39.0%)
VinDr (Test)	8,184	7,228 (88.31%)	956 (11.68%)
Total VinDr + CBIS (Test)	9,587	8,083 (84.31%)	1,504 (15.68%)

The table reports unique image counts per phase and the benign/malignant proportions, highlighting quantity and label skew across clients. During training, 15% of the training set was reserved for validation via stratified sampling.

### 2.2 Preprocessing and augmentation

#### 2.2.1 ROI extraction

For CBIS-DDSM, we used the released lesion-centered crops. For VinDr-Mammo, lesion ROIs were extracted by applying the provided bounding boxes to the original DICOMs; ROIs were exported to 8-bit PNG after windowing the full dynamic range; intensities are preserved up to the applied windowing transformation and only rescaled when forming network inputs. For VinDr-Mammo, the DICOM native photometric VOI LUT was applied when present; otherwise, the full-range window was used.

#### 2.2.2 Label harmonization

VinDr BI-RADS scores were mapped to binary labels: BI-RADS 1–3 → benign (0), BI-RADS 4–6 → malignant (1). Cases with BI-RADS 0 or missing annotations were excluded.

#### 2.2.3 Network inputs

Unless stated otherwise, lesion ROIs were resized to 224×224 px and normalized with ImageNet statistics (mean [0.485,0.456,0.406], std [0.229,0.224,0.225]) to enable transfer learning. We also report a resolution ablation (224→324 px) to assess sensitivity for calcification-rich film data.

#### 2.2.4 Augmentation

To improve generalization: random horizontal flip (p=0.5), random rotation ($\pm 15^{\circ }$), and random Gaussian blur (kernel 5, σ∈[0.1,1.5], p=0.2) were applied during training only.

### 2.3 Learning paradigms

We contrast a conventional *centralized learning* (CL) baseline with simulated cross-silo *federated learning* (FL) under non-IID conditions.

#### 2.3.1 Centralized learning (CL)

All training images from both datasets are pooled; a single model is trained and selected on a held-out validation split.

#### 2.3.2 Federated learning (FL)

**Figure 2:**
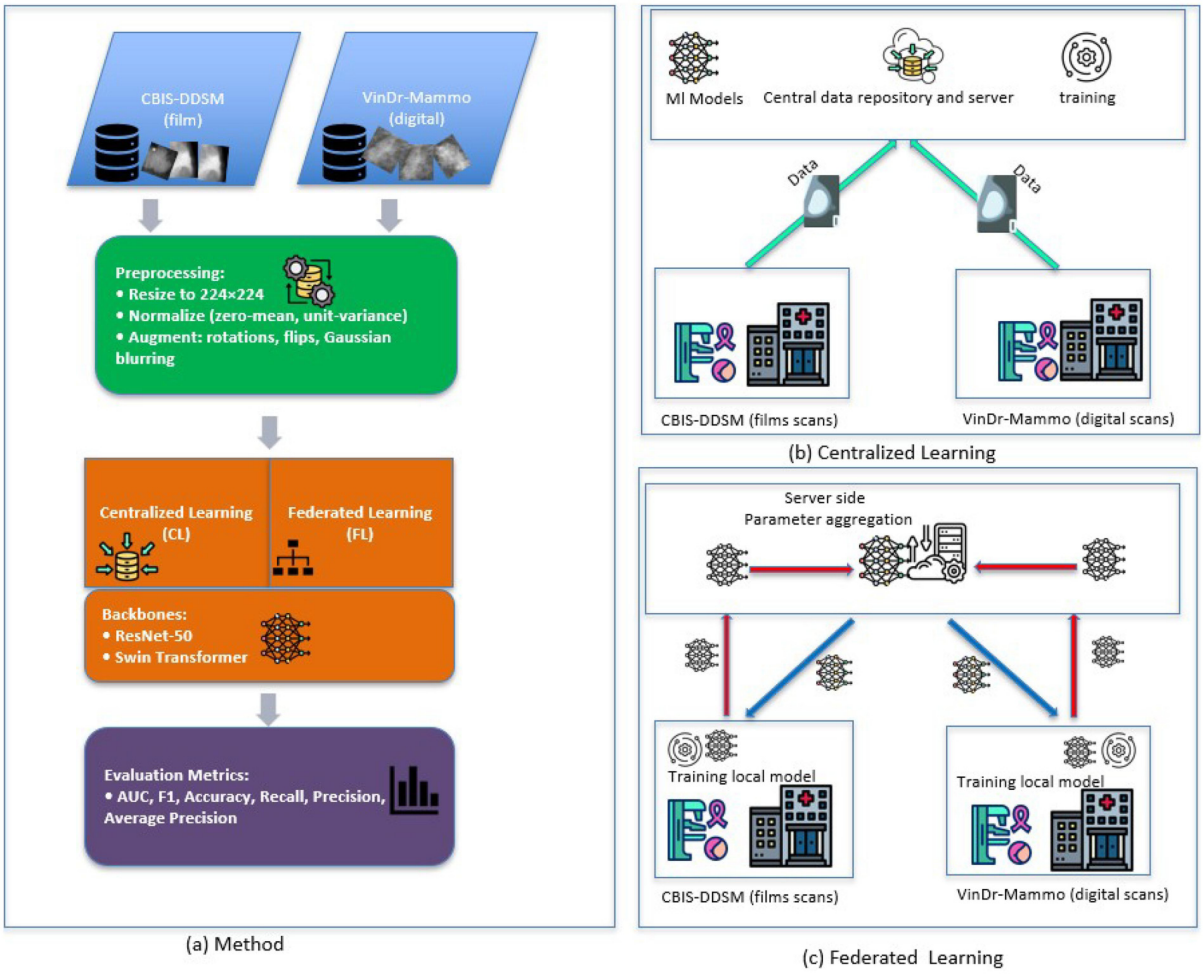
Workflow overview. **(a)** Two data sources (film CBIS-DDSM, digital VinDr-Mammo) feed either a centralized learning (CL) pipeline or a federated learning (FL) pipeline. **(b)** CL pools data for single-site training and validation. **(c)** FL keeps data decentralized across two clients and exchanges only model parameters in synchronous communication rounds, using sample-size–weighted aggregation on a central server.

We simulate two cross-institutional clients CBIS-DDSM (film) and VinDr-Mammo (digital) to explicitly induce feature/quality shift as well as quantity and label skew. Training proceeds in synchronous communication rounds: the server distributes the current global model; each client performs E local epochs on private data; client updates are aggregated to form the next global model. The end-to-end training workflows for CL and FL are summarized in Figure 2.

### 2.4 Federated optimization algorithms

We evaluate four representative algorithms used in cross-silo settings:

- **FedAvg** (9): sample-size-weighted averaging of local model updates; a strong, widely used baseline in medical FL.
- **FedProx** (23): adds a proximal regularizer during local training to reduce client drift under non-IID data; aggregation follows FedAvg.
- **SCAFFOLD** (22): uses control variates to correct client drift; local updates are performed without momentum to align with the control variate formulation; server aggregation remains sample-weighted.
- **FedBN** (24): aggregates only non-Batch Normalization parameters; BN parameters/statistics remain client-specific to preserve domain-dependent feature statistics.

### 2.5 Experimental design and evaluation

#### 2.5.1 Task

Binary lesion classification with predicted malignant probability $\hat{\;p}(y=1|x)$.

#### 2.5.2 Metrics

We report threshold-free ranking metrics (AUROC, Average Precision [AP]), fixed-threshold metrics at τ=0.5 (ACC, Precision, Recall, F1), and a clinically motivated operating point **Precision@Recall = 0.90**. The latter is computed from the PR curve via standard interpolation; bootstrap replicates that cannot attain recall ≥ 0.90 are omitted for that metric (we report the fraction of valid replicates in the supplement when applicable).

#### 2.5.3 Model selection and early stopping

For each experiment, the *test* checkpoint is chosen by *validation AUROC*. If AUROC is undefined in a bootstrap replicate (single-class resample), we fall back to Accuracy. Optional early stopping restores the best validation-AUROC checkpoint.

#### 2.5.4 Bootstrap confidence intervals

We perform n=1,000 example-level bootstrap replicates and report the mean and 95% percentile interval (2.5th–97.5th) for all metrics. Replicates with single-class labels are excluded from threshold-free metrics.

#### 2.5.5 Federated evaluation protocol

For FedAvg, FedProx, and SCAFFOLD, the *single* global model is evaluated on: (i) combined test set and (ii) each domain-specific test set. For **FedBN**, BN buffers are client-specific by design; we therefore inject each client’s cached BN statistics into a clone of the global model prior to evaluating that client’s test data. For the combined test set, each sample is evaluated using the BN statistics of its source domain, and predictions are then aggregated to compute combined metrics.

### 2.6 Implementation details

#### 2.6.1 Software and FL orchestration

Models were implemented in PyTorch. Cross-silo FL was orchestrated in Python using a synchronous parameter-server workflow compatible with standard federated APIs (e.g., FedAvg-style rounds); the training code and experiment orchestration scripts were custom-built for this study.

#### 2.6.2 Backbones and initialization

Based on this prior evidence (15–19), we employed ImageNet-pretrained **ResNet-50** and **Swin Transformer** (Swin V2-T) backbones in all experiments. The original classification heads were replaced with a two-logit fully connected layer suitable for binary classification, while all remaining weights were initialized from ImageNet.

#### 2.6.3 Optimization and precision

Cross-entropy loss; SGD with momentum 0.9, weight decay $1\times 10^{-4}$, learning rate **0.01**, batch size 32. Mixed precision (torch.amp) with gradient scaling on CUDA. For **SCAFFOLD**, local SGD uses *zero* momentum.

#### 2.6.4 Scheduling

Local training uses E=3 epochs per round. We trained for **100** federated rounds for both backbones, guided by pilot curves of validation AUROC.

#### 2.6.5 Training modes

1. **Centralized (CL):** single model on pooled data; selection by validation AUROC.
2. **Local-only:** one model per domain to quantify in-domain vs. cross-domain generalization and the value of collaboration.
3. **Federated (FL):** synchronous rounds with sample-count weighted aggregation; algorithms: **FedAvg**, **FedProx** (μ=0.01), **SCAFFOLD**, **FedBN**.

#### 2.6.6 Data schema and splits

Manifests specify dataset (CBIS vs. VinDr), roi_png_path, and binary label. Validation sets are created via stratified splits with fixed seeds and saved per seed to ensure identical folds across CL, local-only, and FL.

#### 2.6.7 Reproducibility

The code used in this study is publicly available at: GitHub repository. Experiments were run on a single NVIDIA Tesla T4 (16 GB) with cuDNN determinism enabled and benchmarking disabled. We used three fixed random seeds for Python, NumPy, and PyTorch to ensure repeatability.The implementation relies only on standard PyTorch-based server–client abstractions and does not depend on framework-specific features, facilitating reproducibility

### 2.7 Ablations and controls

We include targeted analyses to isolate sources of performance gaps:

- **Size-balancing:** downsample VinDr-Mammo to match CBIS-DDSM to disentangle quantity skew from feature/quality shift (intentionally not class-balanced: all VinDr malignant cases retained; benign subsampled).
- **Homogeneous FL controls:** IID splits within domain (CBIS and VinDr) to determine whether gaps arise from the FL protocol or from cross-domain heterogeneity.
- **Input resolution:** 224 vs. 324 px inputs to test sensitivity for fine-grained calcifications and masses on film.

Dataset statistics for the size-balanced ablation are summarized in Table 2. All ablations follow the same selection, bootstrap, and reporting protocols as the main experiments.

**Table 2:** Ablation dataset statistics for size-balanced training.

Dataset	Images	Benign (0)%	Malignant (1)%
CBIS-DDSM (train pool)	2,301	1,358 (59.0%)	943 (41.0%)
VinDr-Mammo (balanced subset)	2,434	527 (21.7%)	1,907 (78.3%)
Combined (balanced)	4,735	1,885 (39.8%)	2,850 (60.2%)

VinDr-Mammo is downsampled to isolate quantity vs. feature/quality effects.

This ablation is size-balanced across datasets but *not* class-balanced; all VinDr malignant cases were retained and benign cases were subsampled.

## 3 Results

We structure the results according to the four research questions (RQ1–RQ4). All metrics are reported as mean ± SD across three seeds, with 95% bootstrap percentile intervals (1000 replicates). Model checkpoints were selected by validation AUROC.

### 3.1 RQ1: homogeneous performance—how closely can FL match CL when training and testing occur within the same domain?

#### 3.1.1 VinDr-Mammo (digital domain)

In a VinDr-only homogeneous two-client FL control (IID split), FedAvg for ResNet-50 *matched or slightly exceeded* CL (AUC 0.97±0.00 vs. 0.96±0.00; ACC 0.98±0.00 for both), indicating that within-domain FL can approach centralized performance (Table 4). Results from local-only VinDr and heterogeneous FL models on VinDr were consistent. Full CL baselines for both backbones are in Table 3.

**Table 3:** Centralized learning (CL), Local-only, and Federated Learning (FL; FedAvg, heterogeneous two-client CBIS+VinDr) results for ResNet-50 and Swin on CBIS-DDSM, VinDr-Mammo, and the combined test set.

Backbone	Method	Test set	AUC	AP	ACC@0.5	F1@0.5	Precision@0.5	Recall@0.5	Prec@Rec=0.90
ResNet-50	Centralized (CL) baselines								
	CL (pooled)	CBIS	0.70±0.02	0.58±0.03	0.66±0.01	0.55±0.04	0.57±0.03	0.54±0.10	0.45±0.01
	CL (pooled)	VinDr	0.97±0.00	0.94±0.01	0.98±0.00	0.91±0.02	0.96±0.02	0.86±0.03	0.87±0.09
	CL (pooled)	VinDr + CBIS	0.96±0.00	0.88±0.01	0.93±0.01	0.78±0.01	0.81±0.03	0.74±0.02	0.66±0.02
	Local-only baselines								
	CBIS(Local)	CBIS	0.73±0.01	0.63±0.01	0.67±0.01	0.52±0.10	0.61±0.07	0.49±0.18	0.47±0.01
	CBIS(Local)	VinDr	0.53±0.04	0.21±0.04	0.75±0.12	0.23±0.03	0.26±0.19	0.31±0.11	0.11±0.00
	CBIS(Local)	VinDr + CBIS	0.58±0.07	0.31±0.09	0.74±0.10	0.32±0.09	0.32±0.18	0.37±0.07	0.16±0.00
	VinDr(Local)	CBIS	0.52±0.03	0.40±0.01	0.49±0.03	0.48±0.02	0.40±0.02	0.61±0.04	0.39±0.00
	VinDr(Local)	VinDr	0.96±0.00	0.93±0.00	0.98±0.00	0.90±0.01	0.91±0.02	0.89±0.01	0.83±0.07
	VinDr(Local)	VinDr + CBIS	0.93±0.00	0.78±0.01	0.91±0.01	0.72±0.01	0.67±0.02	0.79±0.01	0.51±0.02
	Federated Learning (FL, FedAvg; heterogeneous cross domain one client CBIS and other VinDr)								
	FedAvg	CBIS	0.62±0.01	0.50±0.01	0.49±0.04	0.57±0.01	0.42±0.01	0.86±0.08	0.43±0.00
	FedAvg	VinDr	0.95±0.00	0.89±0.02	0.96±0.01	0.83±0.04	0.88±0.14	0.79±0.05	0.49±0.10
	FedAvg	VinDr + CBIS	0.93±0.00	0.73±0.02	0.89±0.02	0.70±0.02	0.62±0.06	0.82±0.06	0.54±0.04
Swin V2-T	Centralized (CL) baselines								
	CL (pooled)	CBIS	0.61±0.01	0.52±0.03	0.61±0.00	0.16±0.28	0.50±0.50	0.16±0.27	0.41±0.01
	CL (pooled)	VinDr	0.93±0.01	0.82±0.02	0.89±0.02	0.13±0.23	0.33±0.57	0.08±0.14	0.38±0.05
	CL (pooled)	VinDr + CBIS	0.90±0.01	0.60±0.06	0.85±0.01	0.15±0.25	0.55±0.51	0.11±0.19	0.44±0.04
	Local-only baselines								
	CBIS(Local)	CBIS	0.68±0.01	0.60±0.01	0.67±0.01	0.39±0.09	0.71±0.11	0.28±0.11	0.45±0.00
	CBIS(Local)	VinDr	0.66±0.03	0.30±0.02	0.89±0.00	0.16±0.08	0.72±0.19	0.10±0.06	0.13±0.00
	CBIS(Local)	VinDr + CBIS	0.72±0.02	0.41±0.02	0.85±0.00	0.25±0.08	0.71±0.14	0.16±0.08	0.19±0.01
	VinDr(Local)	CBIS	0.48±0.01	0.39±0.01	0.47±0.12	0.38±0.27	0.41±0.05	0.61±0.50	0.38±0.00
	VinDr(Local)	VinDr	0.91±0.02	0.78±0.05	0.86±0.09	0.39±0.32	0.69±0.35	0.50±0.44	0.32±0.07
	VinDr(Local)	VinDr + CBIS	0.87±0.01	0.50±0.03	0.80±0.08	0.38±0.29	0.51±0.16	0.54±0.46	0.39±0.07
	Federated Learning (FL, FedAvg; heterogeneous cross domain one client CBIS and other VinDr)								
	FedAvg	CBIS	0.57±0.03	0.47±0.06	0.47±0.05	0.54±0.02	0.41±0.01	0.80±0.15	0.40±0.01
	FedAvg	VinDr	0.91±0.04	0.75±0.15	0.91±0.02	0.51±0.06	0.84±0.27	0.42±0.17	0.35±0.11
	FedAvg	VinDr + CBIS	0.88±0.02	0.56±0.03	0.85±0.02	0.53±0.04	0.53±0.06	0.56±0.16	0.42±0.09

Here, **CL (pooled)** trains on the full VinDr+CBIS pooled dataset. Metrics are mean ± SD across seeds. CL (pooled) represents the upper bound when all data can be centralized.

#### 3.1.2 CBIS-DDSM (film domain)

Under a homogeneous FL control for CBIS-DDSM, FL closely matched CL (FedAvg AUC 0.75±0.03, ACC 0.70±0.03; CL AUC 0.73±0.01, ACC 0.67±0.01). Local-only CBIS models showed similar in-domain performance, suggesting that FL approximates CL when client distributions are aligned (Table 4).

**Table 4:** Homogeneous two-client FL (FedAvg) vs. CL on CBIS-DDSM and VinDr-Mammo.

Backbone	Method	Test set	AUC	AP	ACC@0.5	F1@0.5	Precision@0.5	Recall@0.5	Prec@Rec=0.90
CBIS-DDSM									
ResNet-50	FedAvg	CBIS	0.75±0.03	0.67±0.02	0.70±0.03	0.60±0.04	0.62±0.04	0.62±0.04	0.59±0.04
	CL (domain only)	CBIS	0.73±0.01	0.63±0.01	0.67±0.01	0.52±0.10	0.61±0.07	0.49±0.18	0.47±0.01
VinDr-Mammo									
ResNet-50	FedAvg	VinDr	0.97±0.00	0.94±0.01	0.98±0.00	0.91±0.02	0.97±0.02	0.86±0.04	0.86±0.06
	CL (domain only)	VinDr	0.96±0.00	0.93±0.00	0.98±0.00	0.90±0.01	0.91±0.02	0.89±0.01	0.83±0.07

Here, **CL (domain-only)** trains only within the domain (CBIS-only or VinDr-only). This table isolates whether gaps arise from the FL protocol or from cross-domain heterogeneity.

#### 3.1.3 Summary

Across both homogeneous domains, FL either matches or closely approaches CL.

### 3.2 Local-only baselines

Local-only models showed strong in-domain performance for both CBIS and VinDr but transferred poorly across domains (e.g., VinDr(Local) → CBIS AUC 0.52±0.03; CBIS(Local) → VinDr AUC 0.53±0.04). These baselines quantify cross-domain difficulty and contextualize FL performance: while FL preserves strong VinDr performance, CBIS remains challenging under cross-domain training. Quantitative results for in-domain and cross-domain transfer are summarized in Table 3.

### 3.3 Comparison of local, centralized, and federated models

**Figure 3:**
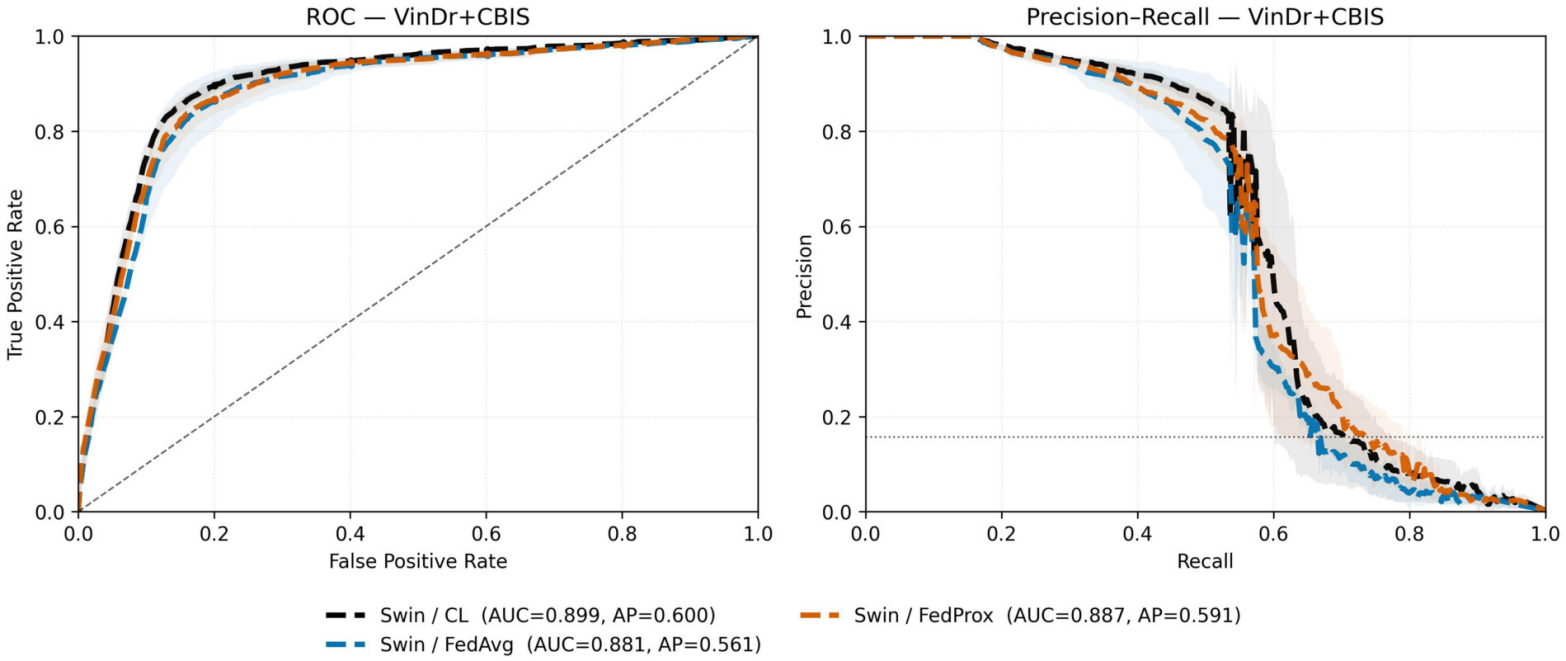
Receiver operating characteristic (ROC) and precision–recall (PR) curves for the **Swin V2-T** evaluated on the **combined VinDr-Mammo+CBIS-DDSM** test set. Methods shown: **CL**, **FedAvg**, and **FedProx**. Curves illustrate ranking performance across operating points; summary metrics (AUROC, AP, Precision@Recall=0.90) are reported in Table 5.

**Figure 4:**
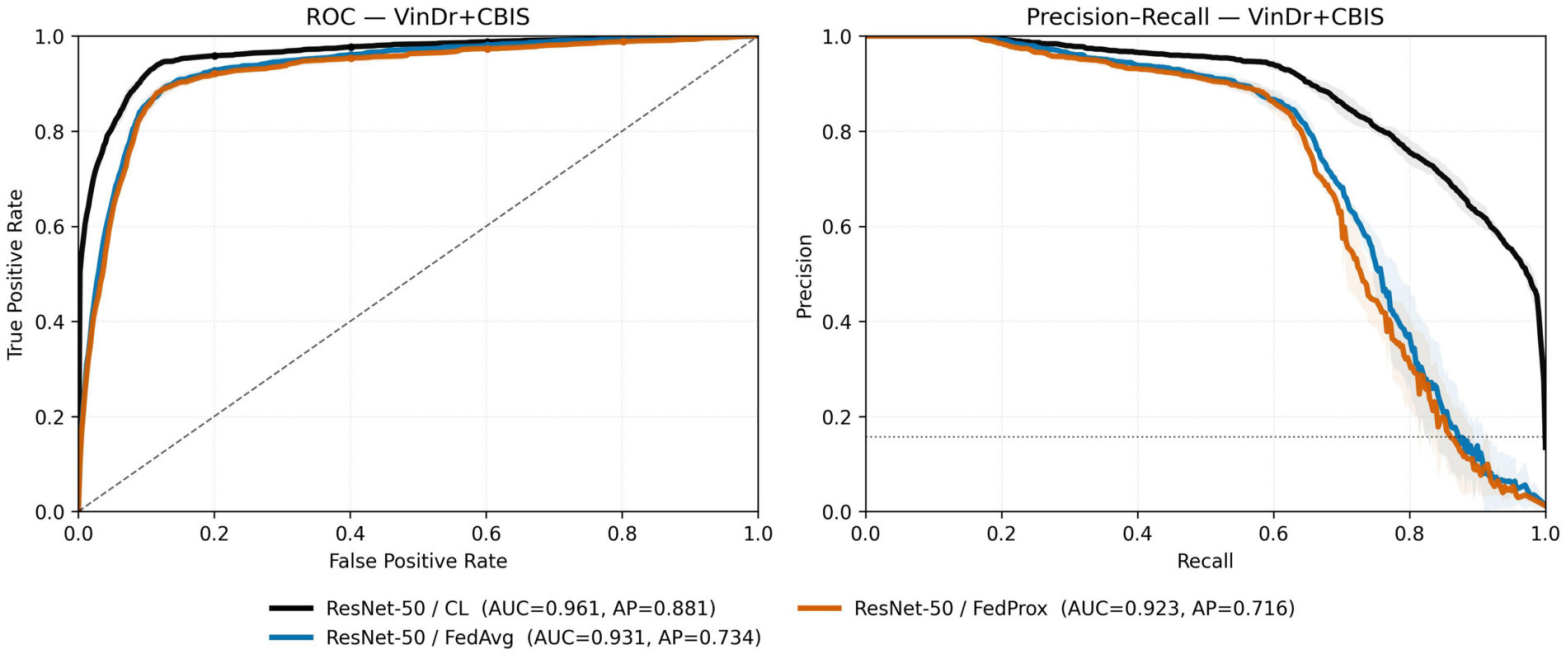
Receiver operating characteristic (ROC) and precision–recall (PR) curves for **ResNet-50** evaluated on the **combined VinDr-Mammo + CBIS-DDSM** test set. Methods shown: **CL**, **FedAvg**, and **FedProx**. These curves complement thresholded operating points (ACC, Precision, Recall, F1 at τ=0.5) in Table 5 and highlight precision–recall trade-offs under cross-domain heterogeneity.

Across both backbones we observe three consistent patterns. (i) In homogeneous settings, FL matches *or slightly exceeds* CL (e.g., VinDr ResNet-50 AUC 0.97 vs. 0.96; CBIS ResNet-50 AUC 0.75 vs. 0.73), confirming that the protocol itself does not reduce performance (Table 4). (ii) FL improves upon local-only models that transfer poorly across domains, demonstrating a clear benefit of collaboration (Tables 3, 5). (iii) Under film–digital heterogeneity, FL performance lies between CL and local-only baselines: it preserves most of the digital-domain accuracy while underperforming on the minority film domain (Tables 3, 5). These trends on the combined test set are visualized by ROC and precision–recall curves comparing centralized learning (CL), FedAvg, and FedProx. For the Swin V2-T backbone, curves are shown in Figure 3; the corresponding ResNet-50 curves are shown in Figure 4.

**Table 5:** Heterogeneous two-client FL results for ResNet-50 and Swin V2-T with FedAvg, FedProx, SCAFFOLD, and FedBN, evaluated on CBIS-DDSM, VinDr-Mammo, and the combined test set.

Backbone	Method	Test set	AUC	AP	ACC@0.5	F1@0.5	Precision@0.5	Recall@0.5	Prec@Rec=0.90
ResNet-50	FedAvg	CBIS	0.62±0.01	0.50±0.01	0.49±0.04	0.57±0.01	0.42±0.01	0.86±0.08	0.43±0.00
	FedAvg	VinDr	0.95±0.00	0.89±0.02	0.96±0.01	0.83±0.04	0.88±0.14	0.79±0.05	0.49±0.10
	FedAvg	VinDr+CBIS	0.93±0.00	0.73±0.02	0.89±0.02	0.70±0.02	0.62±0.06	0.82±0.06	0.54±0.04
	FedBN	CBIS	0.53±0.03	0.42±0.03	0.49±0.08	0.47±0.14	0.39±0.11	0.67±0.31	0.40±0.00
	FedBN	VinDr	0.75±0.21	0.53±0.35	0.66±0.40	0.49±0.28	0.55±0.39	0.71±0.31	0.34±0.18
	FedBN	VinDr+CBIS	0.64±0.16	0.47±0.23	0.57±0.28	0.48±0.20	0.47±0.27	0.69±0.29	0.37±0.13
	FedProx	CBIS	0.61±0.01	0.49±0.01	0.50±0.05	0.57±0.01	0.43±0.02	0.86±0.06	0.42±0.01
	FedProx	VinDr	0.94±0.01	0.89±0.02	0.97±0.01	0.84±0.04	0.95±0.02	0.75±0.06	0.43±0.06
	FedProx	VinDr+CBIS	0.92±0.01	0.72±0.02	0.90±0.00	0.71±0.01	0.64±0.02	0.79±0.06	0.53±0.04
	SCAFFOLD	CBIS	0.48±0.02	0.38±0.00	0.43±0.01	0.51±0.05	0.38±0.02	0.77±0.14	0.40±0.01
	SCAFFOLD	VinDr	0.93±0.01	0.86±0.01	0.95±0.01	0.74±0.06	0.99±0.00	0.59±0.08	0.37±0.04
	SCAFFOLD	VinDr+CBIS	0.90±0.00	0.63±0.02	0.88±0.01	0.62±0.05	0.59±0.01	0.66±0.10	0.48±0.03
Swin V2-T	FedAvg	CBIS	0.57±0.03	0.47±0.06	0.47±0.05	0.54±0.02	0.41±0.01	0.80±0.15	0.40±0.01
	FedAvg	VinDr	0.91±0.04	0.75±0.15	0.91±0.02	0.51±0.06	0.84±0.27	0.42±0.17	0.35±0.11
	FedAvg	VinDr+CBIS	0.88±0.02	0.56±0.03	0.85±0.02	0.53±0.04	0.53±0.06	0.56±0.16	0.42±0.09
	FedBN	CBIS	0.52±0.05	0.42±0.06	0.55±0.08	0.21±0.25	0.24±0.19	0.29±0.40	0.40±0.00
	FedBN	VinDr	0.61±0.31	0.38±0.34	0.64±0.38	0.21±0.24	0.34±0.37	0.36±0.34	0.14±0.02
	FedBN	VinDr+CBIS	0.56±0.20	0.40±0.22	0.59±0.24	0.21±0.23	0.29±0.26	0.33±0.35	0.27±0.13
	FedProx	CBIS	0.58±0.05	0.49±0.05	0.47±0.12	0.37±0.32	0.26±0.23	0.64±0.55	0.40±0.01
	FedProx	VinDr	0.92±0.02	0.79±0.04	0.87±0.09	0.40±0.37	0.41±0.46	0.49±0.44	0.33±0.09
	FedProx	VinDr+CBIS	0.89±0.01	0.59±0.03	0.81±0.08	0.38±0.34	0.30±0.29	0.54±0.48	0.40±0.06
	SCAFFOLD	CBIS	0.53±0.04	0.42±0.05	0.58±0.03	0.11±0.16	0.21±0.19	0.09±0.13	0.40±0.01
	SCAFFOLD	VinDr	0.90±0.02	0.71±0.10	0.88±0.00	0.03±0.04	0.67±0.58	0.01±0.02	0.27±0.02
	SCAFFOLD	VinDr+CBIS	0.87±0.01	0.49±0.02	0.84±0.00	0.07±0.09	0.27±0.24	0.04±0.06	0.36±0.03

Metrics are mean ± SD across seeds. Domain-specific reporting reveals minority-domain degradation that can be masked in combined metrics.

### 3.4 RQ2: heterogeneous two-client FL under film–digital domain shift

Under CBIS+VinDr heterogeneity, global FL models remained strong on VinDr but showed clear degradation on CBIS (ResNet-50 FedAvg VinDr AUC ≈0.95 vs. CBIS CBIS AUC ≈0.62; Table 5). On CBIS, the thresholded operating point at τ=0.5 shifted toward higher recall and lower precision. Among algorithms, FedAvg was the most stable; FedProx produced only marginal changes, SCAFFOLD showed higher variance, and FedBN did not consistently improve minority-domain performance.

### 3.5 RQ3: identifying the source of the performance gap—quantity skew or feature/quality shift?

Two ablation experiments were conducted to isolate the cause of CBIS degradation.

#### 3.5.1 Size-balancing ablation

Equalizing client sizes partially mitigated minority-domain underperformance but did not close the gap to CL and introduced trade-offs on the digital domain. With FedAvg and ResNet-50, CBIS AUC improved from 0.62 (unbalanced FL; Table 5) to 0.68 (balanced FL; Table 7), yet it remained below the balanced CL baseline (0.70). Conversely, VinDr AUC decreased from 0.95 (unbalanced FL) to 0.83 (balanced FL), compared with 0.95 under CL. On the combined test set, balanced FL reached 0.84 AUC vs. 0.93 for CL. Thus, size-balancing improved CBIS but did not match CL, and it reduced VinDr performance, indicating that quantity reweighting alone does not resolve the underlying domain mismatch.

#### 3.5.2 Homogeneous FL controls (CBIS)

In-domain CBIS-Hom FL closely matched CL (AUC 0.75±0.03 vs. 0.73±0.01), indicating that when clients share similar distributions, FL itself is stable and effective. This confirms that cross-domain heterogeneity, not FL protocol instability, is responsible for minority-domain degradation.

#### 3.5.3 Conclusion

Taken together, these ablations show that the principal bottleneck is the film–digital feature and quality shift. Size balancing modifies relative influence but does not resolve the mismatch, and homogeneous CBIS-FL controls confirm that the FL protocol itself remains stable in the absence of cross-domain divergence.

### 3.6 RQ4: operating points and input resolution

Precision@Recall=0.90 indicates consistently strong digital-domain utility but only modest precision on the film domain, regardless of optimizer. Increasing input size from 224 to 324 px consistently improves CBIS calcification F1 with little change in AUROC (Table 6), and the effect persists under size balancing (Table 7), underscoring the value of resolution-sensitive preprocessing.

**Table 6:** Effect of input resolution on CBIS-DDSM for calcifications and masses (mean ± SD across 3 seeds).

Task	Input	AUC	AP	ACC@0.5	F1@0.5	Precision@0.5	Recall@0.5
Calc	224	0.72±0.02	0.62±0.02	0.65±0.02	0.49±0.06	0.60±0.09	0.44±0.12
	324	0.73±0.02	0.64±0.03	0.65±0.04	0.54±0.06	0.59±0.10	0.54±0.16
Mass	224	0.74±0.02	0.66±0.02	0.68±0.02	0.55±0.14	0.63±0.07	0.54±0.24
	324	0.73±0.02	0.65±0.02	0.67±0.04	0.58±0.02	0.59±0.07	0.58±0.10

Increasing input size from 224 to 324 px yields modest, consistent gains for calcification recall/F1 with limited AUROC change.

**Table 7:** Heterogeneous FL (FedAvg) vs. CL on size-balanced training sets for ResNet-50 across CBIS-DDSM, VinDr-Mammo, and combined tests (mean ± SD across seeds).

Backbone	Method	Test set	AUC	AP	ACC@0.5	F1@0.5	Precision@0.5	Recall@0.5	Prec@Rec=0.90
ResNet-50	FedAvg	CBIS	0.68±0.02	0.58±0.00	0.55±0.06	0.60±0.02	0.46±0.04	0.84±0.06	0.45±0.01
	CL	CBIS	0.70±0.03	0.59±0.04	0.60±0.01	0.59±0.03	0.49±0.01	0.75±0.11	0.46±0.02
	FedAvg	VinDr	0.83±0.03	0.63±0.05	0.88±0.01	0.56±0.03	0.50±0.04	0.63±0.03	0.16±0.02
	CL	VinDr	0.95±0.00	0.90±0.01	0.94±0.03	0.77±0.07	0.68±0.11	0.88±0.01	0.58±0.07
	FedAvg	VinDr + CBIS	0.84±0.02	0.60±0.02	0.83±0.02	0.57±0.03	0.48±0.04	0.70±0.03	0.25±0.03
	CL	VinDr + CBIS	0.93±0.00	0.83±0.01	0.89±0.02	0.70±0.03	0.60±0.07	0.83±0.04	0.49±0.03

Size balancing does not eliminate the minority-domain gap, implicating feature/quality shift as the dominant factor.

## 4 Discussion

This work provides a rigorous evaluation of federated learning for mammography lesion classification under strong film–digital domain shift. By combining heterogeneous FL experiments, homogeneous domain analyses, size-balancing ablations, resolution studies, and per-domain evaluation, we provide a granular understanding of FL behavior in realistic multi-site conditions.

### 4.1 FL performs well on homogeneous domains

Across both VinDr (digital) and CBIS (film) domains, FL closely matched centralized learning when the training and testing distributions were aligned. In VinDr, FL retained high accuracy despite heterogeneous training. In CBIS-Hom, FL replicated the CL baseline. These findings suggest that when domain distributions are internally consistent, FL is reliable and effective.

### 4.2 Feature shift—not dataset size—is the dominant bottleneck

Under heterogeneous training, FL models degraded on CBIS while maintaining strong VinDr performance. Size-balancing experiments ruled out quantity skew as a major factor, and homogeneous CBIS-FL controls confirmed FL stability. The primary challenge arises from the substantial feature and quality differences between scanned film and digital mammography, including differences in noise, contrast, resolution, and artifacts that affect lesion appearance. This aligns with the asymmetric performance observed in heterogeneous FL.

### 4.3 Clarifying the relationship between local, CL, and FL models

In homogeneous settings, FL matched or slightly exceeded CL, showing that the protocol itself does not reduce performance. FL consistently outperformed local-only models that transferred poorly across domains, demonstrating a clear benefit of collaboration. Under film–digital heterogeneity, FL performance fell between CL and local-only baselines: it preserved most digital-domain accuracy while underperforming on the minority film domain. Together with the homogeneous controls, these results implicate film–digital feature/quality shift rather than dataset size or federated optimization instability as the primary bottleneck.

### 4.4 Limitations of current FL algorithms under severe heterogeneity

FedAvg proved robust, while FedProx, SCAFFOLD, and FedBN offered no consistent improvements. In our setting, **FedBN did not consistently improve CBIS precision or AUROC**. This pattern aligns with reports that many heterogeneity-aware optimizers require careful tuning and do not reliably outperform FedAvg in medical imaging contexts (25).

More advanced strategies, such as client-specific adapters, partial aggregation, local BN layers, or contrastive alignment, may be necessary to effectively address feature shift.

### 4.5 Clinical interpretation: operating points matter

The CBIS high-recall/low-precision profile reflects a plausible clinical bias toward sensitivity but yields increased false positives, resulting in added workload and potential patient anxiety. Deployment in screening programs should therefore include site-specific threshold tuning, client-level personalization, and continuous post-deployment monitoring. A single universal threshold across heterogeneous sites is not recommended. The discrepancy between ranking metrics (AUC/AP) and fixed-threshold metrics at τ=0.5 on the minority film domain is consistent with probability miscalibration under domain shift: a single global threshold can yield a high-recall/low-precision operating point on CBIS even when ranking is adequate. In practice, we recommend (i) using threshold-independent metrics for model selection and (ii) performing site-specific threshold tuning (or simple post-hoc calibration) before deployment, rather than adopting a universal threshold across heterogeneous sites.

### 4.6 Resolution considerations and implications for practice

Higher input resolution improved calcification detection on CBIS, consistent with the fine-scale texture needed for film-based microcalcifications. When computing power permits, incorporating higher-resolution inputs or multi-scale architectures may improve robustness across mixed imaging cohorts.

### 4.7 Implications for FL consortia

Our findings highlight the need for domain-aware FL algorithms or personalization layers, mandatory per-site performance reporting, site-specific operating point calibration, and resolution-aware preprocessing strategies. The methodological framework used here multi-seed evaluation, bootstrap confidence intervals, per-domain reporting, and ablations provides a reproducible template for assessing FL safety and reliability in clinical imaging collaborations.

## 5 Limitations and future work

This study adopted a two-client configuration to introduce a strong and interpretable domain shift; however, this deliberate simplification does not reflect the full complexity of real-world federated learning (FL) ecosystems, which typically involve numerous clinical sites with heterogeneous hardware, varying connectivity, and inconsistent annotation quality. Modern personalized FL approaches such as feature-alignment with collaborative classifier updates (26) and multi-model cross-aggregation schemes that leverage complementary client representations (27) were not explored here but may provide greater robustness under realistic multi-institutional conditions. In parallel, recent advances in cross-population adaptation using masked autoencoders and contrastive mix-up strategies (28) underscore the importance of explicitly modeling domain divergences across diverse clinical environments. Incorporating these personalization, alignment, and cross-domain representation-learning mechanisms could help overcome the limitations of the current experimental setup and improve generalization in large-scale federated deployments.

Although FL protects raw data, it does not eliminate privacy risks: parameter updates can still be vulnerable to model inversion, membership inference, or reconstruction attacks. Incorporating differential privacy (DP) (29) or secure aggregation via homomorphic encryption (HE) (30) would strengthen guarantees but introduce accuracy and computational trade-offs that require careful exploration. Finally, our resolution choices were limited by compute; future work should assess high-resolution pipelines (e.g., 512–1,024 px inputs) and multi-view integration for full mammographic context.

## 6 Conclusion

Federated learning enables high-performing mammography classifiers without requiring data centralization when training and testing occur within a single, internally consistent domain. Both digital (VinDr-Mammo) and film (CBIS-DDSM) domains demonstrated that FL can closely match centralized learning in homogeneous settings, and CBIS-Hom FL further confirmed that the FL protocol itself is stable when client distributions are aligned.

In heterogeneous two-client configurations, standard FL algorithms remained sensitive to the film–digital divergence. Although global models retained strong performance on the dominant digital domain, they showed reduced precision on film acquisitions, consistent with the feature/quality gap identified in our ablations.

These findings highlight the necessity of domain-aware or personalized FL strategies, per-site threshold calibration, and resolution-aware preprocessing for film-heavy cohorts. The methodology and analyses presented here provide a rigorous framework for evaluating FL under real-world imaging heterogeneity and offer practical guidance for deploying privacy-preserving AI systems in future breast imaging consortia.

## Data Availability

The original contributions presented in the study are included in the article/Supplementary Material, further inquiries can be directed to the corresponding authors.
